# Atrazine Molecular Imprinted Polymers: Comparative Analysis by Far-Infrared and Ultraviolet Induced Polymerization

**DOI:** 10.3390/ijms15010574

**Published:** 2014-01-06

**Authors:** Jun Chen, Lian-Yang Bai, Kun-Feng Liu, Run-Qiang Liu, Yu-Ping Zhang

**Affiliations:** 1 Pesticide Research Institute, Hunan Agricultural University, Changsha 410128, China; E-Mails: junchen713@hist.edu.cn (J.C.); liurunqiang2007@sina.com.cn (R.-Q.L.); 2 School of Chemistry and Chemical Engineering, Henan Institute of Science and Technology, Xinxiang 453003, China; E-Mail: kfliu1988@126.com; 3 Hunan Academy of Agricultural Sciences, Changsha 410128, China

**Keywords:** atrazine, molecular imprinted polymers, far-infrared induced, ultraviolet induced

## Abstract

Atrazine molecular imprinted polymers (MIPs) were comparatively synthesized using identical polymer formulation by far-infrared (FIR) radiation and ultraviolet (UV)-induced polymerization, respectively. Equilibrium binding experiments were carried out with the prepared MIPs; the results showed that MIP_uv_ possessed specific binding to atrazine compared with their MIP_FIR_ radiation counterparts. Scatchard plot’s of both MIPs indicated that the affinities of the binding sites in MIPs are heterogeneous and can be approximated by two dissociation-constants corresponding to the high-and low-affinity binding sites. Moreover, several common pesticides including atrazine, cyromazine, metamitron, simazine, ametryn, terbutryn were tested to determine their specificity, similar imprinting factor (IF) and different selectivity index (SI) for both MIPs. Physical characterization of the polymers revealed that the different polymerization methods led to slight differences in polymer structures and performance by scanning electron microscope (SEM), Fourier transform infrared absorption (FT-IR), and mercury analyzer (MA). Finally, both MIPs were used as selective sorbents for solid phase extraction (SPE) of atrazine from lake water, followed by high performance liquid chromatography (HPLC) analysis. Compared with commercial C_18_ SPE sorbent (86.4%–94.8%), higher recoveries of atrazine in spiked lake water were obtained in the range of 90.1%–97.1% and 94.4%–101.9%, for both MIPs, respectively.

## 1. Introduction

Molecularly imprinted polymers (MIPs) as an artificial template made recognition material with high affinity and selectivity for the target molecule, have attracted more and more attention since this technique was first developed by Wulff [1] and Mosbach [2]. MIPs have been widely applied in many fields including solid phase extraction (SPE) [3,4], chromatography [5,6], enantiomer separation [7], catalysis [8–10] and chemical sensors [11,12]. The potential of MIPs to act as SPE sorbents was first described by Sellergren for the selective determination of metabolites in urine samples [13]. Since this time, various examples of the application of MIPs to the extraction and cleanup of complex samples have been described in the literature [14–16]. The first use of a MIP-based SPE in water analysis was presented by Matsui *et al.* for atrazine and simazine [17]. Prasad *et al.* developed a biomimetic potentiometric sensor by dispersing the atrazine imprinted polymer particles in di-*n*-octyl phthalate plasticizer and embedding it in a polyvinyl chloride matrix. The polymerization process was initiated in an oil bath at 80 °C and heated for about 12 h. The resulting sensor responded to atrazine in the pH range 2.5–3.0 over a wide working concentration range (0.0001–10) mM with a detection limit of 0.5 μM (0.1 ppm) [18]. Guzzella *et al.* developed a propazine MIP for use as a sorbent for SPE of common triazines found in water. This bulk polymerization was carried out over 24 h at 60 °C [19].

Since photo irradiation facilitates homogeneous and rapid heat transfer through the reaction mixture, laboratory photoreactions are increasingly popular in the application of synthetic chemistry. Generally, polymer synthesis by photo irradiation is applicable to most types of polymerization methods, including step growth, free and controlled radical and ring opening polymerizations possible as well as modification and curing reactions [20–22]. Benefits include rapid synthesis, decreased side reactions, higher yields with greater monomer conversion and the ability to utilize “green” solvent systems [23,24]. Imma *et al.* utilised ultraviolet to prepare atrazine MIPs in a photochemical reactor at 350 nm and 4 °C for 16 h by bulk polymerization [25]. Koeber *et al.* prepared terbuthylazine MIPs as SPE materials for environmental analysis, which were similar to formulation by irradiating the solutions with a low-pressure mercury lamp for 24 h [26]. Zhang *et al.* prepared and evaluated melamine molecularly imprinted polymers by thermal-and photo-initiation methods [27]. However, there is little or no research on the mechanism of molecular imprinted polymers by far-infrared polymerization. Differences in physical properties between FIR radiation and UV-induced polymers have been noted, with speed and penetrability observed in photo-initiated free radical polymerization systems [28].

In this work, far-infrared radiation and ultraviolet-induced free radical polymerization methods were applied to prepare atrazine MIPs using methacrylic acid (MAA) as the functional monomer, ethylene glycol dimethacrylate (EDMA) as the crosslinker, dichloromethane as the porogen, 2,2′-azobisisobutyronitrile (AIBN) or Irgacure 1800 as the FIR-radiation initiator or UV-initiator, respectively. Comparative analysis of both MIPs and their respective NIPs were carried out using a variety of instrumental methods (SEM, FT-IR and MA). Binding capacity and imprinting parameters were also evaluated in detail to exhibit differences in selectivity and discrimination for the template and structurally related compounds. The synthesized MIPs were utilized as SPE sorbents for selective extraction of atrazine from contaminated water samples, with sample recovery, followed by the determined by HPLC.

## 2. Results and Discussion

### 2.1. Physical Characterization of the Synthesized Polymers

**Figure 1.**
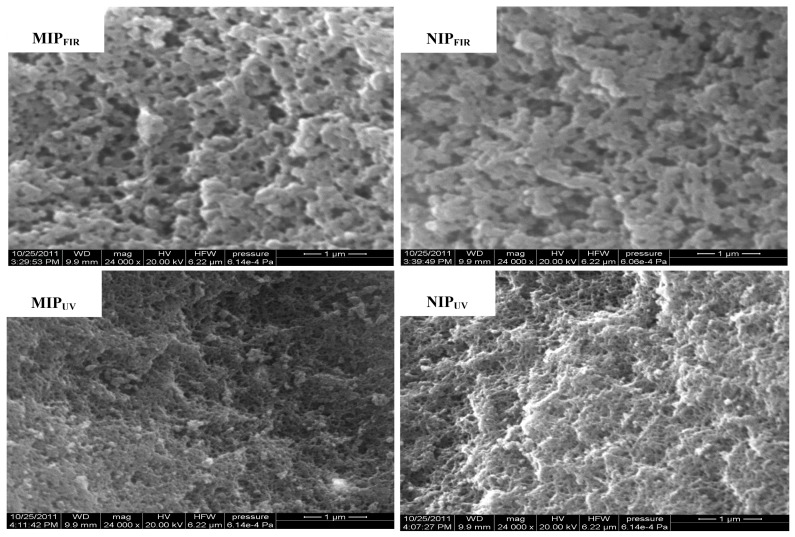
SEM of MIPs and NIPs prepared by FIR- and UV-polymerization methods.

SEM was employed to observe the surface microscopic characteristics of the prepared polymers. Figure 1 shows the distinct differences between two NIPs due to different polymerization methods, which results in corresponding differences between the two MIPs. Regardless whether FIR-or UV-polymerization methods were used, it was shown that MIPs possessed more pores than NIPs and larger average pore diameters were observed for the MIPs. In contrast with MIP_FIR_, MIP_UV_ exhibited significantly smaller average pore diameters and distinctly higher numbers of holes. The pores of the polymers are usually formed by two methods: (1) Large pores are obtained by the presence of porogenic agent (organic solvent); and (2) Cavities are in the imprinting procedure, by providing the specificity by reason of their complementarity towards the template molecules [29].

**Figure 2.**
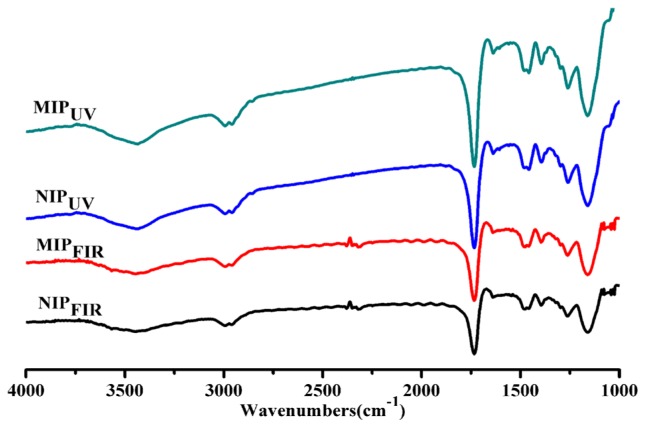
Comparative IR spectra of prepared materials by FIR- and UV-polymerization methods.

FT-IR spectrum is a useful method for characterizing intermolecular hydrogen bonding. The infrared spectra results of all samples were shown in Figure 2. No clear differences in IR bands were observed for the polymer samples prepared by the two different polymerization methods. There were three distinctive absorptions assigned to –COOH groups of the MAA units including C=O stretching vibration at 1702 cm^−1^, O–C–O absorption at 1200 cm^−1^, and –OH stretching vibration at 3440 cm^−1^. For imprinted polymers prepared using MAA as a functional monomer, it was possible that some carboxylic acids changed into dimeric –COOH groups during the imprinting reaction. This means that even though the non-imprinted poly(MAA–EDMA) and the atrazine imprinted poly(MAA–EDMA) contain the same level of functional groups, more carboxylic acids in non-imprinted reference polymers may exist as hydrogen bonded dimers, so that the amount of “free” carboxyl groups in non-imprinted reference polymers becomes lower than the corresponding imprinted polymers. In general, no appreciable band corresponding to the acid dimer can be easily distinguished since the –COOH groups of MAA units associated with –NH groups of the template atrazine via a hydrogen bonding interaction during the polymerization [30]. We noted there were some significant IR bands at 3600–3450 cm^−1^ for both NIPs comparative with MIPs.

**Figure 3.**
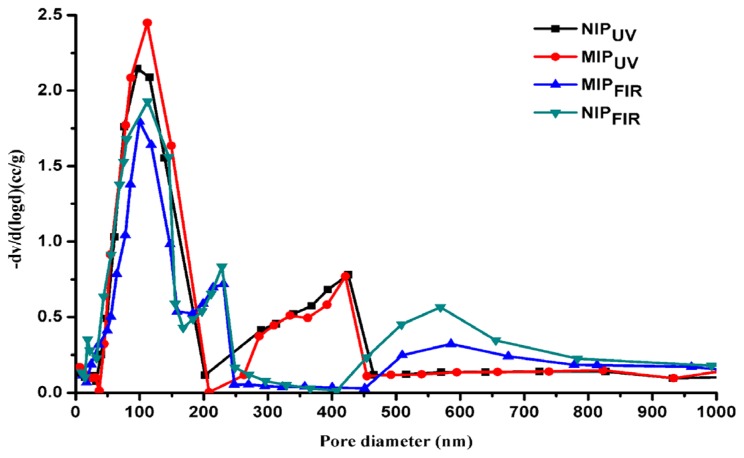
Comparative pore-size distribution of both FIR- and UV-polymerization polymers.

Further analysis of polymer porosity was measured by mercury intrusion porosimetry in Figure 3. It can be seen that medium pores (10–100 nm) and large pores (100–800 nm) account for a large ratio for both MIPs. There were three kinds of pores for MIP_FIR_ and NIP_FIR_ centered at approximately 100, 570, and 220 nm, respectively, with a gradual decrease of peak area accordingly. Whilst two kinds of pores for MIP_UV_ and NIP_UV_ were obtained with a pore size centred about 100 and 420 nm, respectively. Moreover, we note a proportionately greater presence of macropores centred at about 100 nm for MIP_UV_, comparing MIP_FIR_, which accounts for the larger binding amount and surface area for the former. Slight difference of macropores and distinct differences occurred at the macropore level, account for the calculated variations in pore volume and total porosity. The speed of the polymerization process is highly dependent on the number of free radicals that the UV-initiator provides by dissociation under ultraviolet radiation. The faster that polymerization occurs, the greater the degree of conversion of the liquid monomer to a solid polymer. For far infrared thermal polymerization, various absorption energy levels exist for different reactant molecular and transition differences and vary significantly between levels. Hence, the actual absorption is a complex process, accompanied by various absorption energy level transitions. Table 1 lists the comparison of total pore volume, surface area and total porosity between MIPs and NIPs. This indicates that the addition of a template increased the large pores in MIPs so that pore size, total pore porosity and total pore volume in the MIPs is larger than those of the NIPs. Due to its larger surface area, pore volume and total porosity, it is easily understood that better recognition performance for template molecule was obtained for MIP_UV_ in the next absorptive experiments.

### 2.2. Adsorption Capacity of MIPs and NIPs

Equilibrium adsorption experiments were performed to evaluate the binding affinity of MIPs for atrazine. In this experiment, the absorption quantity (*Q*) was calculated by equation as follows:

(1) $$Q=(C_{0}-C)\times V/W$$

Here, *C*_0_ is added the template atrazine concentrations in the solution at initial, *C* is that of free template atrazine in the solution containing imprinted polymer after being shaken for 24 h. *V* and *W* are the volume of bulk solution and the weight of the dry polymer used, respectively. The average data of three measurement results were used for adsorption capacity analysis [31].

**Figure 4.**
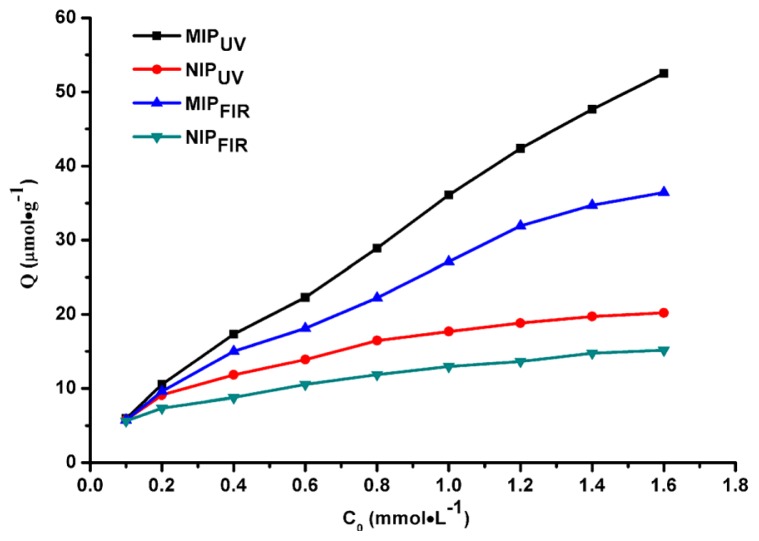
Comparison of equilibrium absorption obtained in separated experiments for both FIR and UV polymerization polymers.

The sorption isotherms of atrazine on the MIP and NIP adsorbents are shown in Figure 4. It can be seen that the sorption of atrazine by the MIPs was significantly higher than that of the respective NIP controls, suggesting an imprinting effect for both MIPs. Template binding for MIP_UV_ exceeded that of MIP_FIR_ across the atrazine concentration range of 0.1–1.6 mmol/L, highlighting MIP_UV_ stronger specific affinity for the target and suggesting the presence of a greater number of more accessible binding sites. Likewise, sorption of NIP_UV_ is correspondingly higher than that of NIP_FIR_, which displayed its stronger non-specific affinity. This reflects the choice of polymerization method and resultant impact on the microstructure of the product polymers. Differences of binding amount could be obtained between the MIPs and their respective NIPs in different ranges of atrazine concentration. When the concentration of atrazine is greater than 0.4 mmol/L, higher differences of binding amount could be achieved for the photo-polymerized materials, compared with the thermal-polymerized materials. For example, the differences of binding amount between MIPs and NIPs are 2.21 and 1.44 μmol/g, for FIR- and UV-induced materials, respectively, when atrazine concentration was equal to 0.2 mmol/L. This difference values were reversed to be 21.3 and 32.3 μmol/g at the concentration of 1.6 mmol/L, suggesting better discrimination between the sites exists in the case of MIP_UV_. This result showed that polymerization of MIP at lower temperatures forms polymers with greater specific affinity for the target *versus* polymers made at elevated temperatures. Usually, the relatively low temperatures with a prolonged reaction time were selected in order to yield a more reproducible polymerization. Where complexation is driven by hydrogen bonding, then lower polymerization temperatures are preferred, and under such circumstances photochemically active initiators may well be preferred as these can operate efficiently at low temperature.

### 2.3. Scatchard Analysis

**Figure 5.**
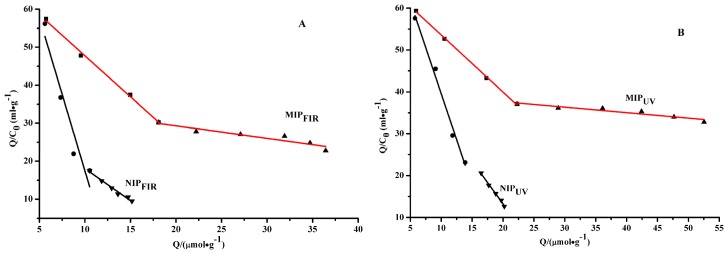
Scatchard plot over 0.1–1.6 mmol/L concentration range for FIR- (**A**) and UV-polymerisation materials (**B**).

If Langmuir models can be applied, the association constant *K*_a_ and specific site capacity *Q* can be determined from the slope and *y* intercept of lines obtained by least-squares regression of linear regions of the corresponding Scatchard plots. Here, the data of the static adsorption experiment was further processed with the Scatchard equation as follows [32]:

(2) $$Q/C=(Q_{\text{max}}-Q)\;K_{\text{a}}$$

where, *K*_a_ is the association constant and *Q*_max_ is the apparent maximum number of binding sites. *C* is the free template atrazine concentration in the solution containing imprinted and non-imprinted polymers. Figure 5 shows saturation binding curves for the atrazine imprinted and non-imprinted polymers by using the saturated binding amount of a template. There are two distinct sections within the plot which can be regarded as straight lines, indicating that the affinities of the binding sites in MIPs are heterogeneous and can be approximated by two dissociation-constants corresponding to the high- and low-affinity binding sites for both polymers, respectively. When the binding amount was small, highly selective imprinted sites contributed largely in static conditions but in chromatographic retention these two types of binding sites acted simultaneously. The above phenomena in the Scatchard analysis are usual for MIPs [33].

The parameters calculated from the curves quantitatively display the differences between two MIPs. The dissociation constants (*K*_a_) and maximum binding amounts (*Q*_max_) for high- and low-affinity binding sites were calculated from the Scatchard equation for the prepared polymers. The respective *K*_a_ and *Q*_max_ values are shown in Table 2. The difference in atrazine binding affinity to the MIPs and NIPs clearly indicated the role of the imprinting process in the formation of specific binding sites. *K*_a_ and *Q*_max_ values for MIP_UV_ were generally more than those of MIP_FIR_ in the studied concentration of template.

### 2.4. Selectivity Experiments for the Prepared Polymers

**Figure 6.**
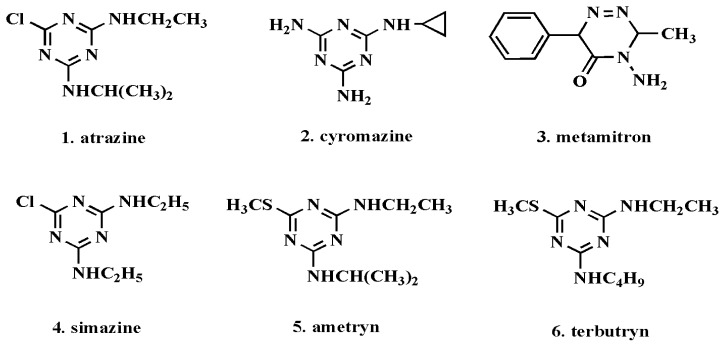
Molecular structure of six analytes for the specificity study.

After equilibrium binding experiments were performed according to the foregoing processes, the relative Q_MIP_ and Q_NIP_ for each analyte could be obtained, respectively. The chemical structures of the studied triazine compounds are illustrated in Figure 6. The imprinting factor expresses the ratio of specific-to-nonspecific binding for each compound (IF = *Q*_MIP_/*Q*_NIP_) [34]. In each case, analyte uptake was normalized against the levels of MIP adsorbed and expressed as selectivity index (SI): SI = I(analyte)/I(template) [35]. These IF and SI values obtained by two methods are given in Table 3.

It can be seen that both MIPs exhibited the highest specificity for atrazine. Furthermore, there is no distinct difference between MIP_UV_ and MIP_FIR_ in recognizing the other five analytes, with similar IF values recorded in each case, but greater SI values were calculated for MIP_UV_ than their MIP_FIR_ counterparts because of comparatively higher levels of specific binding. While atrazine exhibited large retention compared to other tested 1,3,5-triazine derivatives, other structurally different compounds like metamitron showed low-level retention. In IF, atrazine was retained more than other 1,3,5-triazines, such as simazine, terbutryn and ametryn. Therefore it appears that the selectivity of MIPs for atrazine was clearly induced during the imprinting process. The induced recognition ability is particularly noteworthy, because the tested 1,3,5-triazines have only small structural differences. These experimental results demonstrate that imprinting is not only based on the interaction of analyte with functional groups within the three-dimensional polymer network, but also based on the combined effect of shape and size complementarity.

### 2.5. Enrichment of Atrazine Using both MIPs, NIPs and C_18_ as SPE Cartridges

**Figure 7.**
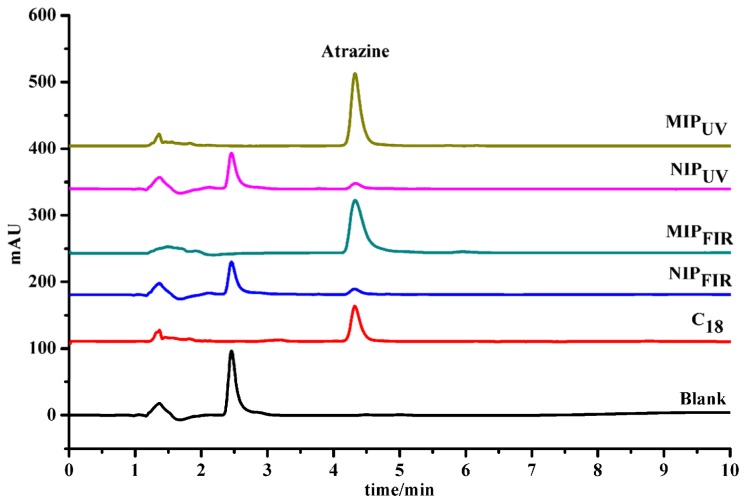
Comparative HPLC chromatograms of the spiked lake water of atrazine with a final concentration 5 mg/L on MIP-SPE and C_18_-SPE columns.

The prepared polymers were compared against commercial C_18_ sorbents as the SPE solid phases to selectively extract atrazine from real water samples. The commercial C_18_ SPE column was successively preconditioned with 3 mL of methanol and 3 mL of LC-grade water. Atrazine solutions of different concentrations were then passed through the columns at a flow rate of 1.0 mL/min, the column was then washed with 3 mL of water containing 5% methanol at the same flow rate. The analyte retained on the polymer was then eluted with 3 mL methanol. These extracts were then evaporated carefully to dryness under a gentle stream of nitrogen at 25 °C, and the residue reconstituted into 3 mL of mobile phase for HPLC analysis. The above procedures were carried out according to the optimized conditions provided by the instruction book of the commercial C_18_ SPE column. Similar procedures for both MIPs and their respective NIPs SPE columns were optimized to pretreat the same lake water, followed by the determination by HPLC. Figure 7 shows that less atrazine was detected in lake water samples following extraction by both NIPs, whilst the atrazine peak sharply increased following extraction by MIP-SPE and C_18_ column separation, respectively. Under the same conditions, different peak area of atrazine was obtained to be 712, 654 and 621, respectively, after the lake water spiked with a final atrazine concentration of 5 mg/L passed through the prepared MIP_UV_, MIP_FIR_ and commercial C_18_ column accordingly. Therefore, higher sensitivities and recoveries could be achieved by using MIP_UV_-SPE as the extraction sorbent. The spiked and determined atrazine were summarized in Table 4. It can be found that the average recoveries of 90.1%–97.1%, 94.4%–101.9% and 86.4%–94.8%, with a RSD lower than 7.49%, respectively. The results demonstrated that MIP_UV_-SPE had highest selectivity and enrichment ability. Hence, the MIP-SPE offers a simple and straightforward technique for direct analysis of atrazine from complicated water samples.

## 3. Experimental Section

### 3.1. Reagents and Materials

MAA, 99.5% and EDMA, 98% were purchased from Beijing Bailingwei Chemical Reagent Co. (Beijing, China) and distilled before use in the up-scaled version of the synthesis. AIBN was obtained from North-China Special Chemical Development Center (Tianjin, China), and recrystallized from methanol before use. Irgacure 1800 was purchased from Ciba Specialty Chem. Inc. (Basel, Switzerland). Pesticide standards: atrazine (98.2%), cyromazine (95.7%), metamitron (95.8%), simazine (96.3%), ametryn (98.4), and terbutryn (96.8%) samples of technical grade were generously provided by China Agricultural University (Beijing, China) and Hunan Agricultural University (Changsha, China). All other chemicals used were chromatographically pure of analytical grade. Distilled and LC grade water was obtained from a super-purification system (Danyangmen Corp., Changzhou, China).

All chromatographic evaluations were performed by a reversed-phase Agilent 1100 HPLC system from the Agilent Company (Santa Clara, CA, USA), containing a quaternary pump. Separation was carried out on a C_18_ chromatography column (150 mm × 4.6 mm i.d., particle size 4 μm, YMC America, Inc., Allentown, PA, USA). Pressure Blowing Concentrator (Supelco, Bellefonte, PA, USA), SPE Manifold (Supelco, Bellefonte, PA, USA), TENSOR27 infrared spectrometer (Bruker, Billerica, MA, USA), PM-33-11 Poremasters (Quantachrome Instruments, Boynton Beach, FL, USA), Quanta200 scanning electronic microscope (FEI, Hillsboro, OR, USA). The UV lamp used in the polymerizations was a medium-pressure mercury vapor lamp (Philips, HPK 125 W). The FIR irradiated using a FIR heater (rated output power at 1000 W, Westa Electric Appliances Co., Ltd., Foshan, China), which emitted radiation at the wavelength range from 10 to 140 μm.

### 3.2. Preparation of Atrazine MIPs by FIR Radiation and UV-Induced Polymerization

**Figure 8.**
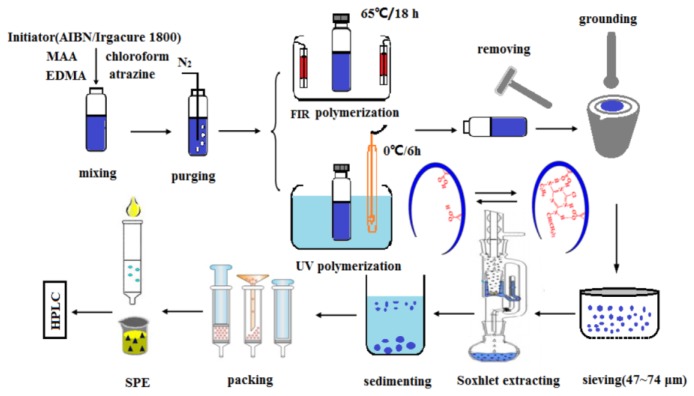
Schematic representative of preparation of atrazine imprinted polymers for SPE.

The synthesis of atrazine-imprinted polymer was similar to that reported in the literature for atrazine [17,20–22]. Briefly, atrazine (1 mmol, 0.215 g) and methacrylic acid (4 mmol, 0.35 mL) were taken in 50 mL ampoule bottle and the mixture was left in contact for 30 min for pre-arrangement. Subsequently, EDMA (20 mmol, 3.78 mL), AIBN (0.24 mmol, 0.043 g) and 5 mL of chloroform were added. The mixture was purged with N_2_ for 10 min and the bottle was sealed under this atmosphere. These samples were then placed in the holding tray in the middle of the FIR heater with rotation for even irradiation at a controlled temperature of 65 °C for 18 h to carry out the polymerization process. In comparison, 0.04 g photo-initiator (Irgacure 1800) was added instead of AIBN in the above solutions using the identical polymer formulation, but the sealed tubes were irradiated with a UV lamp (intensity, 0.016 W/cm^2^) at ice bath 0 °C for 6 h. After polymerization, the polymerization tubes were crushed, and the polymers were removed, then ground and sieved. Particles between 47 and 74 μm were collected and then repeatedly suspended in acetone to remove the small particles. Non-imprinted polymers (NIPs) were synthesized under the same conditions but without the addition of the template. The template was removed from the MIPs by Soxhlet extraction using a two step procedure: The mixture solution of acetic acid and methanol (1:9, *v*/*v*) was firstly used for washing about 12 h, followed by methanol for 6 h as a second step. This procedure was repeated four times. The extracts were analyzed by LC/VWD until a stable baseline was obtained. The prepared polymers were dried at 60 °C for 24 h under vacuum and stored for the next experiments. The overall process for the preparation of MIPs is depicted in Figure 8.

### 3.3. Preparation of SPE Cartridges Using the MIPs and NIPs as the Sorbents

Briefly, a 60 mg dry polymer was fully mixed and packed into empty SPE cartridges of 3 mL between two frits (length of 65 mm and i.d. 10 mm, DIKMA Sci. & Tech., Beijing, China). The cartridges were subjected to vacuum for 30 s before insertion of a second frit on top of the sorbent bed. Four different cartridges using both polymers as the sorbents were prepared according to the above method in our experiments.

### 3.4. Affinity and Specificity Study of Both MIPs

In order to investigate the binding property of the resulted polymers, static absorption experiment and Scatchard analysis were employed in this work. In vials, the polymer particles 20.0 mg were mixed with 2.0 mL of various concentrations of atrazine (from 0.1 to 1.6 mmol/L) in chloroform. The mixture was shaken at 120 rpm in a thermostatic shaker at 25 °C for 24 h. These solutions were centrifuged and filtered, then followed by the determination of the free concentrations of atrazine by HPLC at 220 nm. Each binding amount was determined in triplicate and calculated based on the standard curve. Single-analyte binding experiments of other similar pesticides including cyromazine, metamitron, simazine, ametryn and terbutryn were also performed using the above method to investigate the specificity of MIPs for atrazine.

### 3.5. MIP-SPE for Atrazine Standard Solutions and Real Samples

Before loading analyte, the MIP-SPE column was previously conditioned with 5 mL of methanol and LC-grade water, successively. After atrazine standard solutions at different concentrations were passed through the columns at a flow rate of 1.0 mL/min, the columns were washed with 6 mL of water and 2 mL of methanol at the same flow rate. The analyte retained on the sorbent was eluted with 5 mL methanol containing 5% ammonium hydroxide. These extracts were evaporated carefully to dryness with a gentle stream of nitrogen at 25 °C, and the residue was reconstituted into 1 mL of mobile phase for further HPLC analysis.

Once the optimized MIP-SPE experimental conditions were established, lake water spiked with atrazine at five concentration levels of 0.5, 1, 5, 10 and 20 mg/L, respectively, was used to demonstrate the applicability of the resulted MIPs to pre-concentration of atrazine from the real samples. Briefly, three bottles of 2 L water samples were collected from sampling sites in the East Lake of the Henan Institute of Science and Technology, and delivered to the analytical laboratory stored at 4 °C in a refrigerator. Static settlement and filteration of lake water samples should be dealt with prior to sample analysis. After the SPE cartridge was conditioned, then 5 mL of real sample was passed through. Finally, the eluate was dried using an N_2_ stream followed by adding the mobile phases with a final constant volume of 5 mL.

HPLC conditions employed for this work were as follows: mobile phase, ethanol/water (60:40, *v*/*v*); flow rate, 1.0 mL/min; room temperature; UV detection at 220 nm; injection volume 20 μL.

## 4. Conclusions

UV-induced bulk polymerization of atrazine imprinted polymer results in a 3-fold reduction in preparation time compared with conventional FIR thermal initiation methods. The resultant MIP_UV_, which behaved different gross morphologies to their FIR counterparts, exhibited higher binding capacity to their counterparts MIP_FIR_. Furthermore, the high and low affinity sites present on MIP_UV_ possessed significantly larger *K*_a_ and *Q*_max_ values than those of MIP_FIR_, indicating better discrimination between the sites exists in the case of MIP_UV_. MI-SPE-HPLC was successfully applied to the extraction and determination of atrazine in lake water and gradual increase of recovery for the determination of atrazine in lake water was, respectively, obtained using C_18_ SPE sorbent, MIP_FIR_ and MIP_UV_ accordingly.

## Figures and Tables

**Table 1.** Physical properties of the MIPs and NIPs determined by MA.

Polymer	Total pore volume (cm^3^/g)	Surface area (m^2^/g)	Total porosity (%)
MIP_FIR_	0.44	198.92	64.58
NIP_FIR_	0.39	145.74	52.93
MIP_UV_	0.52	213.92	83.64
NIP_UV_	0.48	179.25	77.13

**Table 2.** The adsorption parameter of MIPs and NIPs.

Polymer	Linear regression equation		*K*_a1_ × 10^−4^ (mol/L)	*Q*_max1_ (μmol/g)	*K*_a2_ × 10^−4^ (mol/L)	*Q*_max2_ (μmol/g)

	High-affinity	Low-affinity				
MIP_FIR_	*Q*/*C* = 66.39 − 1.86Q	*Q*/*C* = 35.23 − 0.31*Q*	5.38	35.70	32.30	113.93
NIP_FIR_	*Q*/*C* = 117.13 − 10.89Q	*Q*/*C* = 34.95 − 1.69*Q*	0.92	10.76	5.93	20.70
MIP_UV_	*Q*/*C* = 67.23 − 1.37Q	*Q*/*C* = 40.26 − 0.13*Q*	7.33	49.25	76.50	307.78
NIP_UV_	*Q*/*C* = 83.02 − 4.41Q	*Q*/*C* = 54.04 − 2.04*Q*	2.27	18.96	4.90	26.49

**Table 3.** Maximum binding number (*Q*_max_), imprinting factors (IF) and standardized selectivity index (SI) for MIP_FIR_, MIP_UV_, NIP_FIR_ and NIP_UV_ (*n* = 6).

Substrate	*Q*_MIP_ (μmol/g)		*Q*_NIP_ (μmol/g)		IF		SI	
			
	FIR	UV	FIR	UV	FIR	UV	FIR	UV
atrazine	27.12	36.12	12.96	17.47	2.09	2.07	1.00	1.00
cyromazine	20.71	28.57	12.43	17.64	1.67	1.62	0.74	0.79
metamitron	12.02	14.29	10.85	11.25	1.11	1.27	0.49	0.62
simazine	18.55	25.52	11.59	15.66	1.60	1.63	0.71	0.79
ametryn	15.13	23.87	11.07	16.13	1.37	1.48	0.61	0.72
terbutryn	14.01	22.61	11.31	16.87	1.24	1.34	0.55	0.65

**Table 4.** Recoveries of atrazine obtained from spiked lake water samples.

Polymer	Standard addition amount (mg/L)	Determined (mg/L)	Recovery rate (%)	RSD (*n* = 3) (%)
MIP_FIR_	0.5	0.47	94.7	5.28
	1	0.97	97.1	5.12
	5	4.89	97.8	4.85
	10	9.08	90.8	3.27
	20	19.21	90.1	2.76

MIP_UV_	0.5	0.47	94.6	7.13
	1	0.95	95.3	4.52
	5	4.92	98.4	4.13
	10	9.44	94.4	3.47
	20	20.37	101.9	5.92

C_18_	0.5	0.45	90.3	7.49
	1	0.86	86.4	3.78
	5	4.56	91.2	4.26
	10	8.95	89.5	2.51
	20	18.96	94.8	2.17
